# Examining the postural awareness and flexibility changes in physical therapy students who took clinical Pilates class

**DOI:** 10.12669/pjms.333.12808

**Published:** 2017

**Authors:** Esra Atilgan, Devrim Tarakci, Fatma Mutluay

**Affiliations:** 1 Esra Atilgan, PT, PhD. Department of Physiotherapy and Rehabilitation, Istanbul Medipol University Faculty of Health Sciences, Istanbul, Turkey; 2 Devrim Tarakci, PT, PhD. Department of Ergotherapy, Istanbul Medipol University Faculty of Health Sciences, Istanbul, Turkey; 3 Prof. Fatma Mutluay, PT. Department of Physiotherapy and Rehabilitation, Istanbul Medipol University Faculty of Health Sciences, Istanbul, Turkey

**Keywords:** Clinical Pilates, Education, Flexibility, Postural Awareness

## Abstract

**Objective:**

This study aimed to evaluate postural awareness and changes in posture and flexibility of students who took Clinical Pilates class as an elective course at the undergraduate level.

**Methods:**

The study conducted from 2013-2016 included 98 students who took Clinical Pilates class at the Department of Physical Therapy and Rehabilitation, School of Health Sciences, Istanbul Medipol University, Turkey. The flexibility levels of the study participants were measured before and after the Clinical Pilates education using finger-to-floor test and modified Schober’s test. Observational posture analysis and postural awareness were recorded using the scale prepared by the researchers.

**Results:**

The post-education evaluations showed that postural distortions were fixed, and a significant increase in the postural awareness of the students was observed compared with the pre-education period. The results of both modified Schober’s test and finger-to-floor test, which were used to measure the flexibility levels, showed a statistically significant increase in post-education scores compared with those of the pre-education period.

**Conclusion:**

This study showed that the Clinical Pilates course increased postural awareness and flexibility of physical therapy students and fixed postural distortions. Thus, the inclusion of Clinical Pilates class in the undergraduate education is considered to be important.

## INTRODUCTION

The Clinical Pilates method was developed by Joseph Pilates in the early 1900s and called “Contrology.” This method began to be used by dancers in the 1980s. Then, it attracted the attention of health care providers in the 2000s and began to be used in different disease groups for rehabilitation purposes apart from the healthy individuals.^1^-^4^

Clinical Pilates has eight basic principles: concentration, breathing, centering, control, precision, flowing movement, isolation, and routine. Proper use of Pilate’s principles ensures core stabilization. It also improves static and dynamic balances through posture, flexibility, and postural awareness.^2^,^5^,^6^

Previous studies showed that Clinical Pilates exercises reduced pain, improved life quality, and increased functional capacity. Short-term Clinical Pilates education was found to have an effect on body composition.^7^,^8^

These studies also showed that Clinical Pilates education was customized in different cases, such as low back and neck pain, postural distortions, orthopedic injuries, neurological problems, rheumatic problems, cancer, osteoporosis, osteoarthritis, scoliosis, and pregnancy apart from the cases of healthy individuals.^2^,^5^,^9^,^10^

During the physical therapy undergraduate education, basic lessons, such as exercise physiology and exercise education, are taught intensely. However, Clinical Pilates education is not included intensely in the curriculum programs. Therefore, the Clinical Pilates method has been taught to physical therapists through the postgraduate education practices in recent years. It is important for physical therapists to learn Clinical Pilates for both patients and their own body images due to the ever-increasing popularity of this method. This case should be taken into consideration during the revision of the physical therapy education.

The pilot study of the present research conducted in 2015 showed that Clinical Pilates course provided in a small group increased postural awareness and had positive effects on posture.^11^ Therefore, with the support of the previous studies and the present study, the researchers planned to point out the Clinical Pilates education by examining postural awareness, posture, and postural parameter changes in physical therapy students.

## METHODS

**Fig.1:**
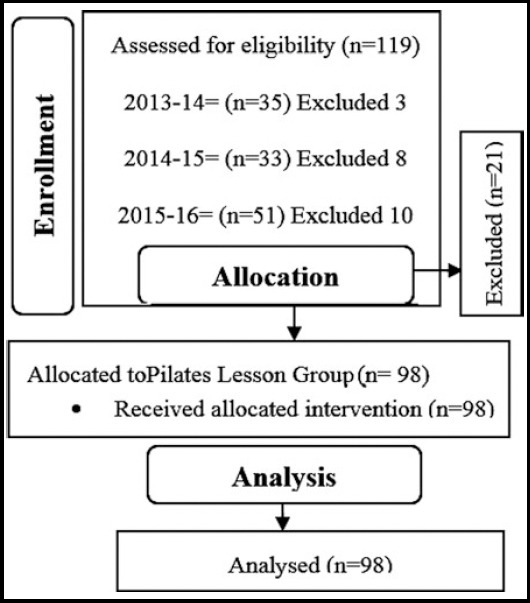
Flow diagram of the study

The study included the students who took Clinical Pilates class at the Department of Physical Therapy and Rehabilitation, School of Health Sciences, Istanbul Medipol University, Turkey in 2013−2014, 2014−2015, and 2015−2016 academic years and voluntarily participated in the study (Fig.1). The present study was approved by the Istanbul Medipol University Non-Interventional Clinical Studies Ethics Committee with the decision no. 10840098-320.

The Clinical Pilates course was conducted with a maximum of 30 people two times a week for 14 weeks to enrich exercise prescriptions for diseases by providing the final-year students with awareness education.

Before the students were provided with basic knowledge of the Clinical Pilates course, the first evaluations were made. They were informed about the Clinical Pilates exercises within the scope of 14-week education, and the exercises were performed practically. All evaluations were repeated at the end of the semester.

### Posture Evaluation

The participants of the study were observed from the anterior, posterior and lateral perspectives for postural properness and the specified postural distortions were recorded.^12^

### Flexibility Evaluation

The flexibility levels of the study participants were measured before and after the Clinical Pilates education using the modified Schober’s test and finger-to-floor test, which were included in the flexibility measurements.

### Lumbar Flexion Measurement

The lumbar flexion was measured using the modified Schober’s test. The distance between the superior and inferior parts of the posterior iliac spine was marked for measurement. The researchers identified 10 cm above and 5 cm below of the marked field. The patient was asked to lean forward. The variance between this position and the initial value was written down in centimeters. A variance of 0–5 cm in the test indicated a decreased flexion level, while a variance of 10 or above indicated an increased flexion level. Values between 5 and 10 cm were considered to be normal.^13^

### Finger-to-Floor Test

The body flexion was evaluated using the finger-to-floor test. The test was performed ensuring that an individual stood on a block with a height of 15 cm, leaned forward without bending knees, and tried to touch on fingertips. The block was identified as the “0” point and the participant were asked to cross the block stretching out the arms forward. The distance between the individual’s middle finger and the block was measured. The obtained result was recorded in centimeters. If the participant managed to cross the block by stretching out, the plus (+) score was given. If the individual failed to cross, the minus (–) score was given.^12^

### Postural Awareness

Postural awareness was evaluated before and after the Clinical Pilates education. Here, the individuals were asked to verbally evaluate their own postures before and after the Clinical Pilates education. The evaluation was conducted using a numeric analog scale including 0–10 points that were prepared by the researchers. In this scale, the point “0” meant very poorpostural awareness and the point “10” meant high postural awareness (Table-I).

**Table-I:** Postural awareness form.

0	1	2	3	4	5	6	7	8	9	10
0:	Bad Postural awareness							
1-2-3:	Mild Postural awareness					7-8-9:	Good Postural awareness				
4-5-6:	Modarete Postural awareness					10:	Excellent Postural awareness				

### Statistical Analysis

The data were evaluated using the Statistical Package for Social Science (SPSS) version 21.0 software for Windows and by analyzing descriptive statistics (frequency, mean, and standard deviation (SD). The Mann-Whitney U Test was used to determine the effects of the Pilates education program. A significance level of 0.05 was used.

## RESULTS

The present study included 98 students who continued their final year in the Physical Therapy undergraduate program and whose mean age was 21.98±1.03 years (Fig.1). The participants’ sociodemographic data are given in Table-II.

**Table-II:** Baseline socio demographic and clinical characteristics of students.

Students(n)	98	
Female n(%)	86(87,5%)	
Male n(%)	16(12,20%)	

		*Min-max*

Age (x±SD, years)	21,98±1,03	21-26
BMI(x±SD, kg/m^2^)	21,16±2,85	15,79-32,69

The pre-education evaluations showed that the most commonly observed postural distortions in physical therapy students were rounded shoulders (27%), increased lordosis (21%), kyphosis (11%), and head tilted forward (10%). A postural correction was observed after the Clinical Pilates education (Table-III).

**Table-III:** Changes within groups after pilates course.

	*Baseline*	*After14 weeks*		

*Postural changes*	*n (%)*	*n (%)*		
Anterior tilt of head (%)	10(%10,2)	5(%5,51)		
Rolled of shoulder (%)	27(%27,6)	9(%9,2)		
Kyphosis (%)	11(%11,2)	6(%6,1)		
Anterior pelvic tilt (%)	21(%21,4)	7(%7,1)		

	*(x±SD)*	*(x±SD)*	*p(2 tailed)*	*t*

Flexibility Measurement				
Modified Schober test (cm)	6,62±3,17	7,35±3,05	0,001	-3,344
Finger ground test (cm)	2,91±4,61	6,44±5,93	0	-6,472
Postural awareness(0-10)	4,08±2,45	8,57±10,09	0	-3,86
p<0,05				

The results of both modified Schober’s test and finger-to-floor test, which were used to measure the flexibility levels, showed a statistically significant increase in post-education scores compared with those of the pre-education period (p<0.05) (Table-III). A statistically significant increase in the students’ postural awareness levels was observed compared with the pre-education period (p<0.05) (Table-III).

## DISCUSSION

Physical therapy education includes the basic exercise education and the exercise physiology, anatomy, and biomechanics classes. Physical therapists use the therapeutic effects of exercises in different disease cases. The fact that Clinical Pilates exercises have been included in the rehabilitation field in recent years led the physical therapists to receive postgraduate Clinical Pilates education and begin specializing in this field.

Clinical Pilates, which has been used as a therapeutic exercise, provides a proper posture by enabling the deep postural muscles to be strengthened because it is an exercise system that helps body’s postural muscles to function in a balanced manner, supports the spine, and raises the spine’s awareness in its neutral position.^2^,^5^,^11^

Studies have shown that Clinical Pilates exercises improve flexibility, activate transverse abdominis, provide lumbo-pelvic stability, and enhance muscle activity of healthy individuals.^14^ Significant changes were observed as a result of the modified Schober’s test and finger-to-floor test because Clinical Pilates exercises included flexibility exercises for muscles and were performed in different planes raising the awareness.

A previous study examined the Clinical Pilates exercises that were performed once a week for two months by the healthy individuals and followed up for six months. It revealed that the exercises improved the flexibility (p<0.01) and the posture, but did not statistically change the body mass index.^15^ The present study, on the contrary, examined different effects and revealed significant changes in postural awareness and flexibility. This dissimilarity was supposed to result from both the extended period and the differences in Clinical Pilates performance methods.

Although postural distortions, such as head forward, increased lordosis, rounded shoulders, and kyphosis, were highly observed in most of the individuals before the education, these distortions significantly decreased after the education. Kucuk et al.^16^ reported that the belief in exercise, self-esteem, and body perception improved as a result of the Clinical Pilates education conducted three times a week for 8 weeks. These changes in posture are attributed to the belief in exercise and professional respect that are important for physical therapists.

Previous studies showed that Pilates exercises performed by healthy individuals for 12 months corrected the postural alignment, enhanced the balance^17^, and improved life and sleep quality.^18^ Clinical Pilates exercises, performed particularly by healthy groups, have protective effects on health, indicating the importance of these exercises.

A previous study examined the Clinical Pilates exercises performed by young girls for four weeks. It emphasized that the girls considered Clinical Pilates to be entertaining and showed that these exercises could be used in case of obesity, although to a lesser extent.^8^ Despite the fact that the class provided in the present study was an elective course, a great majority of the participants were female students. Moreover, performing Clinical Pilates exercises was entertaining and enabled postural changes, which was similar to the finding of the aforementioned previous study.

A previous study that was conducted with university students included Pilates mat and Tai Chi Chuan exercises during a class period (15 weeks). These exercises were observed to improve the sleep quality and mental health parameters, and no significant change was found in balance and muscular force.^19^ Unlike this study, significant changes were observed in the present study in parameters such as postural awareness and flexibility after the education period. This case is attributed to the fact that the Clinical Pilates class was provided for physical therapy students, and the education period was longer in the present study.

A study conducted by Caldvell et al.^20^ included students and showed that the Clinical Pilates method practiced regularly by the students who received dance training three times a week during a semester raised awareness and enhanced well-being. Regarding the professional working conditions of physical therapists, Clinical Pilates is considered to be important for raising awareness and preventive health care because body mechanics like dancers are used.

Due to the wide use of the media and Internet in Turkey, incorrect and inadequate use of Clinical Pilates exercises has increased, and therefore musculoskeletal problems have been commonly observed. Clinical Pilates has been recently included in the undergraduate education of physical therapists, and its use in rehabilitation has become quite popular nowadays. This exercise method, not so dissimilar to the physical therapy because it is accordant with the treatment principles in Turkey and grounded on anatomy and biomechanics concepts, forms a safe exercise modality when used in a wide variety of fields by specialized physical therapists.

In conclusion, it was observed that depending on the education that was given, the postural awareness of physical therapy students increased, their flexibility improved, and postural distortions were prevented after the Clinical Pilates class. The fact that Clinical Pilates education fixed postural distortions of physical therapy students is important for them to set an example for their patients. Therefore, the inclusion of Clinical Pilates class in the undergraduate education is considered to be important.

### Authors Contribution

**EA, DT and FM** conceived, designed and did statistical analysis & editing of manuscript.

**EA, DT and FM** did data collection and manuscript writing.

**EA, DT and FM** did review and final approval of manuscript.
